# How can citizen science enhance mental health research quality: theory of change development

**DOI:** 10.1136/bmjopen-2024-091007

**Published:** 2025-09-25

**Authors:** Olamide Todowede, Stefan Rennick-Egglestone, Doreen Boyd, Stuart Moran, Andy Bell, Angela Sweeney, Akiko Hart, André Tomlin, Dan Robotham, Julie Repper, Kara Rimmer, Mark Brown, Mark Howells, Shuranjeet Singh, Paula Lavis, Fred Higton, Corrine Hendy, Mike Slade

**Affiliations:** 1 Institute of Mental Health, University of Nottingham, Nottingham, UK; 2 School of Social Policy and Society, University of Birmingham, Birmingham, UK; 3 Centre for Mental Health, London, UK; 4 Kings College London, London, UK; 5 NSUN, London, UK; 6 National Elf Service, London, UK; 7 McPin Foundation, London, UK; 8 ImRoC, Nottingham, UK; 9 Social Spider, London, UK; 10 Nottinghamshire Healthcare NHS Foundation Trust, Nottingham, UK; 11 Taraki, London, UK; 12 NHS Confederation, London, UK; 13 NDORMS, University of Oxford, Oxford, UK; 14 Nord University, Bodo, Norway

**Keywords:** MENTAL HEALTH, PUBLIC HEALTH, Community Participation

## Abstract

**Abstract:**

**Objective:**

Public involvement in mental health research enhances research quality. The use of citizen science methods in mental health research has been described as a conclusion of a movement towards increased public involvement; however, this field is in its early stages of development. Our objective was to create a theory of change (ToC) for how citizen science can be used to enhance mental health research quality.

**Design:**

Iterative consultation with the stakeholders of an existing citizen mental health science study, that is, change for citizen science to achieve co-production at scale (C-STACS: https://www.researchintorecovery.com/research/c-stacs/)

**Methods:**

We co-developed a ToC through an iterative consultation with C-STACS stakeholders who were (a) representatives of mental health community organisations (n=10), individuals with public involvement experience (n=2) and researchers (n=5). In keeping with established ToC practice, entities were identified, including long-term impacts, outcomes needed to create an impact, stakeholder assumptions and indicators for tracking progress.

**Results:**

A desired primary long-term impact of greater co-production of research was identified between researchers and members of the public, which would create a secondary impact of enhancing public capacity to engage in citizen mental health science. We proposed long-term outcomes needed to enable this impact: (1) greater co-production of research objectives and pathways between researcher and the public, (2) greater embedment of citizen mental health science into funder processes (eg, the creation of specific funding calls for citizen mental health science proposals, (3) greater clarity on the boundaries between citizen science and other participatory approaches (eg, so that there is not loss of impact due to conceptual confusion between these, (4) increased knowledge around effective frameworks to enable mass public participation and (5) greater availability of technology platforms, enabling safe and accessible engagement with citizen mental health science projects.

**Conclusion:**

The proposed ToC is grounded in the C-STACS project, but intended to be broadly applicable. It allows the continued formation of a community of practice around citizen mental health science and should be reviewed, as greater knowledge is developed on how citizen mental health science creates change.

**Strengths and limitations of this study**

- This study explored the use of citizen science in mental health research with a range of mental health stakeholders and researchers and some with lived experience of mental health challenges.
- We outlined a theory of change for citizen science to achieve co-production at scale through consultation rounds, which included academics, mental health stakeholders and people with lived experience of mental health.
- Qualitative methods were used to synthesise the outcomes of the consultations and define the impact and outcomes expected from using citizen science in mental health and develop a theory of change.
- The theory of change is built on stakeholders assumptions; therefore, further iterations are required to improve its application to the development and implementation of future mental health citizen science projects.

## Introduction

 Mental ill health is a global crisis. A meta-analysis of papers included in a systematic review estimated that 14.3% of all deaths each year are attributable to diagnosable mental disorders.^1^ In England, the number of people living with mental health challenges has risen, with a 33% increase in referrals to mental health services from 2019 to 2023, and a further projected increase by 2030.^2 3^ Although research can generate evidence relevant to mental healthcare practice, and hence contribute to efforts to address the global mental health crisis, continued innovation is needed to bridge known gaps between research and practice, such as evidence-based treatments not making their way into routine practice.^4^

Many countries now have a substantial tradition of patient and public involvement (PPI) in mental health research. Effective PPI is an important component of work to bridge the gap between research and practice because it can enhance the quality and appropriateness of research^5^,^7^ and enhance our understanding of mental health.^8^,^12^ PPI is enacted through a wide range of working practices, such as the co-production of knowledge between researchers and members of the public.^13 14^ The health research system of the UK has taken a lead in enabling PPI, including resourcing activities and establishing supportive policy frameworks.^15 16^ In 2021, the UK National Institute for Health and Care Research (NIHR), a public research funder of research, endorsed a briefing note advocating involvement of ‘people with lived experience, whether current patient or not’.^17^ This is important when most people experiencing mental health challenges are not in contact with statutory services.^18^

PPI can also directly support the quality of health service provision,^19^ at *micro-level* (eg, in individual care decision-making, planning and management), *meso-level* (eg, in local service planning, monitoring and evaluation, advocacy, training and recruitment of staff, input into guidelines) and *macro-level* (eg, policy making, national-level planning and advocacy).^12 20 21^ PPI in mental health has run in parallel to research in which people who publicly acknowledge personal lived experience of mental health challenges take leadership, for example, in peer-led and mental health survivor research.^22^ Challenges to public participation in PPI practices remain, such as ensuring mutual benefit^23^ and avoiding extractive processes.^24^ PPI barriers have been identified through scoping reviews of existing research. These include token involvement, use of medical terminology during involvement activities, power inequalities between researchers and the public and unequal representation of marginalised and minoritised groups.^12 25^ Active planning is needed to overcome challenges and barriers and enable PPI to make an active contribution to system transformation.

Citizen science is an approach to engaging the general public in science, which involves active participation in science processes at all levels of engagement, from the formulation of the research question and design to data collection, analysis and dissemination.^26^ It has become widely used in fields, such as biology, conservation and ecology,^27^ where it is often used to address real-world research questions, build community capacity for science participation, shape policy decision-making and foster cross-disciplinary knowledge production.^28^ It is beginning to be applied in health research, for example, to identify and prioritise community-focused solutions to chronic disease prevention^29^ and understand how social and environmental issues affect mental health and well-being at a population level.^30^ Citizen science practices already encompass multiple levels of public involvement; hence, these practices are of interest to PPI practitioners. In mental health, the application of citizen science may contribute to mental health system transformation and has been described as a conclusion of the movement towards increased public engagement in research.^31 32^

A 2023 systematic review demonstrated that citizen science has been used in mental health; however, its application was limited; only nine articles were included, despite extensive searches.^33^ A 2024 report found a greater range of in-progress projects, suggesting a growing community of practice.^34^ This report identified two distinct types, defined by form of public engagement: contributory citizen mental health science, where researchers initiate and design research projects, and citizens are involved in mass data collection or analysis, and co-created citizen mental health science, where citizens work together with researchers to jointly design and carry out research projects, which can be led by researchers or citizens or by both working in equal partnership. Citizen science project designers influence the depth of public engagement and involvement in research,^26 35^ and the optimal level of involvement is dependent on project objectives, design and resources.^26^ Despite increasing attention to using citizen science in mental health research, further work is needed to evaluate the feasibility of applying it at scale and evaluate its impacts on stakeholders.^29 36^

Citizen mental health science as a field is in its early stages of development. We currently lack an understanding of how to design and conduct influential citizen mental health science projects. This study seeks to describe how the citizen science approach can be used to enhance mental health research quality, where our notion of quality is broadly defined, to encompass desirable qualities such as active engagement and partnership between researchers and the public, including people with mental health lived experience.

## Methods

We co-produced a preliminary theory of change (ToC) by describing how incorporating citizen science methods into mental health can enhance research quality. Co-production was done through a structured consultation process attended by a range of stakeholders with relevant expertise. Because citizen mental health science is in its early stages of development, this ToC will be refined by others, hence the reason why we refer to it as a preliminary ToC. A structured consultation, rather than (for example) an empirical analysis of existing projects, was chosen because of the limited number of citizen mental health science projects that have been reported.

ToC development was conducted by the citizen science to achieve co-production at scale (C-STACS) project (www.researchintorecovery.com/research/c-stacs). C-STACS planned a systematic review on citizen mental health science^33^ and citizen science projects to (a) identify self-management strategies that people affected by mental problems find helpful and (b) generate visions for a recovery-supporting mental health system. Findings are forthcoming.

C-STACS has defined citizen science as a practice of public participation and collaboration in all aspects of scientific research to generate and increase knowledge and build trust among researchers, policymakers and community members,^37^ operationalised by the 10 European Citizen Science Association (ECSA) principles of citizen science, which enable the development and implementation of good practices in citizen science and are sufficiently flexible for application across diverse situations and disciplines.^38^ Our project sought to virtually engage and generate new knowledge with many public members, referred to as *citizen scientists*. This term is unrelated to legal citizenship status.^39^

With the University of Nottingham research ethics committee, we confirmed that ethical approval for the consultations was not required by our institutional code of research conduct, as stakeholders were collaborators in a co-production process rather than *research participants*.^40^ This is in keeping with the ECSA principle 1, that is, citizen scientists may act as contributors, collaborators or project leaders and have a meaningful role in the project.^38^

### Patient and public involvement

C-STACS was delivered by a consortium of mental health researchers, public advisers and public organisations with knowledge and interest in improving mental health, who participated in the C-STACS Advisory Board, with a remit to advise and support the successful project implementation. All advisory board members were stakeholders in the development process of the ToC. We focused on developing a ToC grounded in the ambitions of C-STACS but transferable to other citizen mental health science projects.

### Theory of change methodology

A ToC is a knowledge product that describes how a specific initiative might create desired changes and for whom. ToCs are typically developed through an iterative approach with expert contributors.^41^ If developed early in a project, ToC can guide project planning, implementation and evaluation,^42^,^44^ for example, by drawing attention to the outcomes that should be assessed in an evaluation. For this paper, this definition of ToC was adopted: “…… predictive assumptions about the relationship between desired changes (ie, *active engagement with people with lived experience of mental health*) and the actions that may produce those changes (ie, *the use of citizen science in mental health*)”.^44^

In developing this ToC, our structured consultation followed established practices^45^,^47^ of (a) defining the real-world impact that we aimed at, (b) deciding on the long-term outcomes and outcomes preconditions and defining the causal pathways between them, (c) adding the necessary interventions or activities required to achieve these outcomes, (d) adding assumptions or rationales to the causal pathways and (e) defining indicators of success for each outcome. Additionally, issues specific to citizen mental health science projects were identified, and their influence was considered on the intended broader social impact.

**Figure 1:**
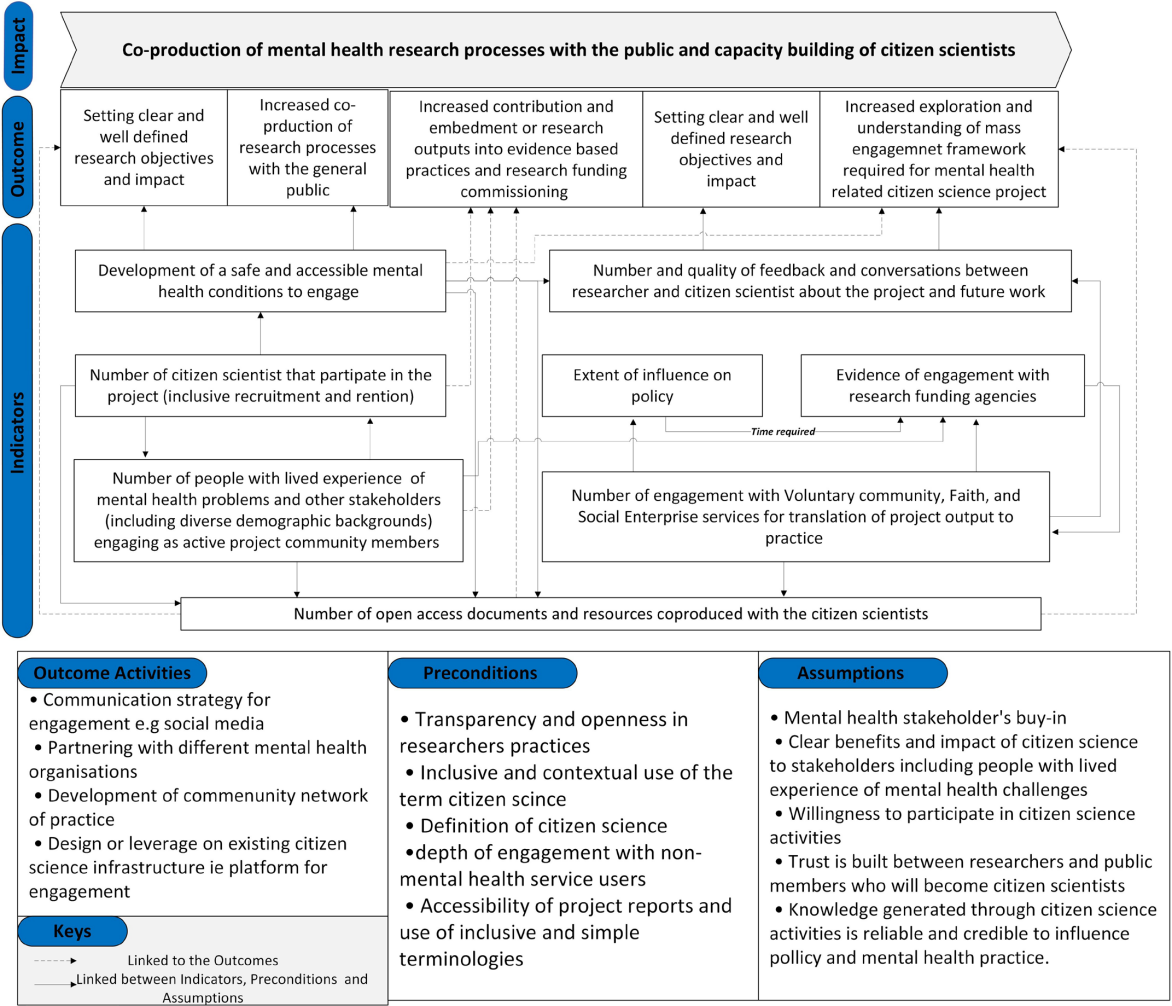
Theory of change map underpinning the C-STACS project. The figure illustrates the key inputs, activities and expected outcomes that guide the project’s design and delivery. C-STACS, citizen science to achieve co-production at scale

Within this structure, impact is a form of long-term ambition for a research programme, which often occurs long after the programme is completed.^48^ The long-term outcomes comprise the expected changes to be observed as a result of the research programme.^49^ The assumptions are the conditions under which the ToC will work, and the indicators are the conditions for measuring the progress of each outcome.^48^ Preconditions are short and intermediate outcomes that may be required to achieve long-term outcomes.^42^ Our final ToC was presented as a map, illustrating the relationship between entities (figure 1). We followed the checklist for reporting ToC in public health interventions^47^ (online supplemental appendix A).

### Stakeholders

The stakeholders were the C-STACS advisory board, composed of (a) C-STACS researchers and investigators (DB, MS, OT, SM and SRE), (b) two people with substantial mental health involvement experience as facilitators or members of the NIHR Nottingham Biomedical Research Centre involvement team (CH and FH) and (c) representatives of organisations with an interest in mental healthcare and policy. DB and SM have led citizen science projects in earth science and ecology, and OT, MS and SRE have developed effective PPI practices, including through a prior study that won a national award for its PPI work (https://institutemh.org.uk/news/1531-neon-trial-wins-2022-mental-health-research-service-user-and-care-involvement-award). At least six members publicly acknowledge personal experience of common or severe mental health problems.

We worked with stakeholders who were representatives of organisations with viewpoints on the mental healthcare system and mental health policy. These organisations are listed in table 1.

**Table 1:** Organisational stakeholders in our theory of change consultation

Organisation	Focus
Centre for Mental Health https://www.centreformentalhealth.org.uk/	Challenging injustices in policies, systems and society
Imroc https://www.imroc.org/	Transforming mental health systems, services and communities so that they are focused on recovery and living well.
McPin Foundation https://mcpin.org/	Enabling people with relevant personal experience to be involved in research
The Mental Elf https://www.nationalelfservice.net/mental-health/	Public dissemination of mental health evidence
National Survivor User Network https://www.nsun.org.uk/	Network of people and groups with lived experience of mental ill-health, distress and trauma, which works to shift power and resources in mental health
NHS Confederation https://www.nhsconfed.org/	Membership organisation that brings together, supports and speaks for the whole healthcare system in England, Wales and Northern Ireland
Nottinghamshire Healthcare NHS Foundation Trust https://www.nottinghamshirehealthcare.nhs.uk/	Provider of intellectual disability, mental health, community health, forensic and offender healthcare services across Nottinghamshire, Leicestershire, Lincolnshire and South Yorkshire
Service User Research Enterprise (https://www.kcl.ac.uk/research/sure	Academic research group comprised predominantly of researchers with direct experience on neurodiversity, trauma, violence and abuse, mental distress and/or (ref)using mental health services
Social Spider CIC https://socialspider.com/	Socially-minded research and project development community interest company (CIC).
Taraki https://www.taraki.co.uk/	Mental health in Punjabi communities.

### Design and procedure

Seven stakeholder consultations were held between August 2022 and July 2023. Participation in the consultation was voluntary, with stakeholders participating as and when they were able. No participation incentives were given, and all consultations were held online. The details of the attendance and length of each consultation, purpose and structure are outlined in table 2.

**Table 2:** Structure of consultations used to develop the theory of change

	Activities	Duration	Number of participants	Focus of consultation	Structure
1	1st online group consultation	10–25 min	11	Identifying possible impacts of using citizen science in mental health	An interactive session through Miro interactive boards
2	2nd online group consultation	10–25 min	9	Determining desired outcomes	An interactive session through Miro interactive boards
3	3rd online group consultation	10–25 min	10	Determining the assumptions	An interactive session through Miro interactive boards and email
4	4th online group consultation	10–25 min	11	Determining the indicators and preconditions	An interactive session through Miro interactive boards and email
5	1st online discussions	60 min	15	Development of the ToC Map, causal pathways and stakeholders checking and editing the summary presentation of the earlier four online group consultations report	Focus group discussion on Microsoft Teams and through email
6	2nd online discussions	60 min	16	Consensus and approval of the ToC map and processes, opportunity for stakeholders to identify and contribute new inputs that they consider relevant to the ToC. For example, stakeholders identified complex issues that might occur while developing and implementing mental health citizen science research	Focus group discussion on Microsoft Teams and email correspondence
7	Consultation with public members	45 min	2	Testing out the assumptions	Discussion on Microsoft Teams

The virtual group consultations with stakeholders were conducted through Microsoft Teams (https://www.microsoft.com/en-gb/microsoft-teams) for live discussions, during which participants collaboratively developed and agreed on components of the ToC using an interactive Miro board (https://miro.com). Records of both were retained.

Meetings were facilitated by the lead researcher (OT). Agendas were structured to enable stakeholders to discuss and reach a consensus on the various ToC elements (example in online supplemental appendix B). The nominal group technique, a structured method that encourages equal participation and helps reach consensus by generating, was used by ranking and prioritising ideas, during the consultations to support decision-making among stakeholders.^50 51^

### Analysis

After each consultation, the lead researcher (OT) independently examined all records and updated the emerging ToC map, which defined the entities in the ToC. This was supported through regular meetings with MS to discuss data interpretations and critical findings. While consultations frequently involved co-producing map updates during the consultation, the additional time taken to reflect on the content of these meetings frequently led to the identification of additional candidate enhancements. These were then examined during a member checking process in a subsequent consultation meeting,^52^ in which proposed ToC map enhancements were discussed and agreed. Therefore, the final ToC map should be seen as a co-produced artefact, which adequately represents the consensus of those consulted.

## Results

Table 3 summarises the entities identified in our ToC process.

**Table 3:** Entities identified in the theory of change

1. Impact	
1.1	Primary: greater co-production of research between researchers and members of the public.
1.2	Secondary: citizen scientist capacity building.
2. Outcomes	
2.1	Greater co-production of research objectives and pathways between the researcher and the public.
2.2	Greater embedment of citizen mental health science into funder processes (eg, the creation of specific funding calls for citizen mental health science proposals).
2.3	Greater clarity on the boundaries between citizen science and other participatory approaches (eg, so that there is no loss of impact due to conceptual confusion between these).
2.4	Increased knowledge around effective frameworks to enable mass public participation (such as designs for projects that can be replicated and reused).
2.5	Greater availability of technology platforms enabling safe and accessible engagement with citizen mental health science projects.
3. Indicators	
3.1	General recruitment and retention of citizen scientists
3.2	Recruitment of citizen scientists with personal experience relevant to mental health.
3.3	Number of projects delivered through a safe and accessible citizen mental health science platform.
3.4	Amount of contribution made to mental health policy.
3.5	Degree of influence over research funding agencies, quality of engagement with agencies that can translate project output to practice.
3.6	Number of open access documents and resources coproduced with the citizen scientist and number and quality of feedback conversations between researcher and citizen scientist about the project and future work.
4. Assumptions	
4.1 Population level	
4.1.1	Large numbers of people are willing to participate in mental health citizen science.
4.1.2	Stakeholders with relevant characteristics are willing to buy into the concept of citizen mental health science.
4.1.3	The distinctiveness of stakeholder contributions could be understood by projects.
4.1.4	Stakeholders could perceive potential benefits from citizen science projects.
4.2 Community level	
4.2.1	Relevant communities will trust researchers to engage with them.
4.2.2	Researchers are willing to actively engage with and be sensitive to community needs.
4.3 Policy level	
4.3.1	Mental health citizen science activities and knowledge generated are accepted as credible by stakeholders engaged in policy development work.
4.3.2	The knowledge generated by citizen mental health science influences mental health policy and systems, including enabling funding agencies to invest in citizen mental health science projects.
5. Preconditions (all projects)	
5.1	Transparency and openness on the part of researchers.
5 Preconditions (relevant to specific types of projects).	
5.2	Inclusive use of terminology.
5.3	Depth of engagement with non-users of mental health services.
5.4	Inclusive language and accessible technology.
6. Outcome activities	
6.1	Communication strategy for engagement.
6.2	Partnering with different mental health organisations.
6.3	Development of a community network of practice.
6.4	Design or leverage of existing citizen science infrastructure.

These entities were defined as follows.

### Impact

Stakeholders agreed that the primary desired long-term impact of integrating the citizen science approach into mental health was the greater co-production of research between researchers and members of the public. More distal impacts, such as increased public engagement, were considered, but they have greater dependencies on other forms of societal change and hence are less amenable to control by the public or research community. Citizen scientist capacity building is a likely consequence of this primary impact and, hence, was classified as a secondary impact, with a range of benefits to these individuals and wider society.

### Outcomes

Generating these primary and secondary impacts will require a wide range of changes (referred to as outcomes). We enumerated the four most critically important outcomes required to create long-term impact while acknowledging these as a subset of the outcomes required to enable impact. These included 1) greater co-production of research objectives and pathways between researcher and the public, (2) greater embedment of citizen mental health science into funder processes (eg, the creation of specific funding calls for citizen mental health science proposals,; (3) greater clarity on the boundaries between citizen science and other participatory approaches (eg, so that there is not loss of impact due to conceptual confusion between these, (4) increased knowledge around effective frameworks to enable mass public participation (such as designs for projects that can be replicated and re-used), and (5) greater availability of technology platforms enabling safe and accessible engagement with citizen mental health science projects.

However, outcome 4 recognises that frameworks that have been successfully used to enable mass participation in domains such as ecology may only be partially transferable to citizen mental health science, due to challenges such as greater ethical concerns surrounding mental health data than those in ecology. This means that existing mass participation frameworks may need to be modified, or new frameworks need to be developed, for successful use in citizen mental health science.

### Indicators

As for outcomes, the most critical indicators to measure project success were identified. These included (i) general recruitment and retention of citizen scientists, (ii) recruitment of citizen scientists with personal experience relevant to mental health (eg, lived experience of mental health challenges, lived experience of mental health support, lived experience of minoritisation, the latter because experiences of minoritisation predict mental health problems), (iii) number of projects delivered through a safe and accessible citizen mental health science platform, (iv) amount of contribution made to mental health policy, (v) degree of influence over research funding agencies, (vi) quality of engagement with agencies who can translate project output to practice, (vi) number of open access documents and resources co-produced with the citizen scientist and (vii) number and quality of feedback conversations between researcher and citizen scientist about the project and future work. Stakeholders noted that changes, such as policy influence, can take both specific planning and greater time to enact, which must be adjusted for during project evaluation.

### Assumptions

Stakeholders agreed that assumptions in the ToC could be identified at three levels: *population, community and policy*. The population-level assumptions were as follows: (i) large numbers of people are willing to participate in mental health citizen science, (ii) stakeholders with relevant characteristics are willing to buy in to the concept of citizen mental health science (eg, service users, carers, healthcare staff and policymakers), (iii) the distinctiveness of stakeholder contributions could be understood by projects and (iv) stakeholders could perceive potential benefits from citizen science projects. The *community-level* assumptions were as follows: (i) relevant communities will trust researchers to engage with them, and (ii) researchers are willing to actively engage with and be sensitive to community needs (especially for communities experiencing inequality due to racism, low socio-economic status and digital access). The *policy level* assumptions included the following: (i) mental health citizen science activities and knowledge generated are accepted as credible by stakeholders engaged in policy development work, and (ii) the knowledge generated by citizen mental health science influences mental health policy and systems, including enabling funding agencies to invest in citizen mental health science projects.

### Preconditions

Stakeholders saw transparency and openness on the part of researchers as an essential precondition, as researchers worked with citizen scientists. This included clarifying the values and benefits of citizen science to mental health research and differentiating between citizen science and other participatory research approaches that are commonly used in mental health. The latter will reduce incorrect assumptions about the project process and outcomes and manage expectations between intended citizen scientists and researchers.

Other preconditions were identified for specific forms of projects, as shown below:

1. **Inclusive use of terminology:** The term citizen science may be insufficiently inclusive in projects drawing in people not identifying or accepted as a citizen of a country (eg, refugees or asylum seekers). *Community science* or *community mental health knowledge* might be interchangeable terms for *citizen science*.
2. **Depth of engagement with non-users of mental health services:** Since most people experiencing even severe forms of mental health problems are not engaged with statutory health services, for many projects, engagement with people not using services should be seen as a precondition for generating useful knowledge.
3. **Inclusive language and accessible technology:** The use of accessible language and technology will help the public understand the complexity of the project and its possible impact. That reduces the fear and concerns of prospective citizen scientists about mental health by providing the meanings of any terminology and explaining the importance of the citizen science approach.

### Complexity of conducting mental health citizen science research

Stakeholders identified three specific considerations for citizen mental health citizen science projects that were not formally part of the stakeholder map:

1. Findings from the mental health citizen science project should not solely focus on informing clinical practice but also consider a holistic approach to engaging and informing the general population, including those who are not using mental health services.
2. The use of citizen science approaches to encourage stakeholder engagement in mental health systems, including research, is important. However, engagement in research and discussions with members of the public should address the relationship between social issues and mental health outcomes, as this is currently not addressed within patient and public involvement approaches. Furthermore, the expertise and experiences of people with lived experiences of mental health must be respected, supporting involvement in shared decision-making and preventing tokenism. This takes time and resources, which are often limited during research project implementation.
3. A citizen science approach is commonly used within the wellness industry for commercial purposes, *for example, the generation of mental health fitness data from the public through digital watches or applications*. Therefore, the use of the citizen science approach in research for mental health engagement, such as the C-STACS project, must be clearly defined. This emphasises the complexities, pluralities and varied individual mental health experiences. These complexities and purpose of citizen science mental health projects must be acknowledged throughout the development and reporting.

## Discussion

In this study, a ToC was developed to enhance knowledge and inform researchers and mental health stakeholders on how to build citizen science projects for mental health by actively engaging the general population. We believe that this is the first application of established procedures for ToC development to understand how citizen mental health science can create change. Citizen science has emerged as a valuable approach to working with the general population to better understand health problems and promote community engagement.^29 53^ Our ToC provides a process and impact framework to guide the implementation and evaluation of future mental health citizen science projects, incorporating the rationale behind the approach and contextual influences and addressing barriers to implementation.^47^ The ToC developed in this study is intended for application to a range of mental health-related citizen science projects, but was developed through the expertise of the C-STACS project.

Our stakeholder consultations concluded that a viable long-term impact for the mental health citizen science projects was greater co-production of research between researchers and members of the public, which would build citizen scientist research capacity as a secondary impact. This is likely to occur through community building, active engagement and equal partnership between the public, including people with lived experience of mental health, researchers, policymakers and other interested mental health stakeholders. The long-term outcomes required to create these impacts included increased clarity between the intersection of citizen science and other participatory research approaches and increased understanding of the framework required to engage a large number of public members in crowdsourced/citizen science mental health-related research.

This study offers insights into the application of citizen science in mental health research to understand the nuances between the use of the citizen science approach and other participatory research methods that are commonly used in the mental health field. The use of this approach in mental health is sparsely reported and limited to its use in participatory data collection.^33^ Mental health research is known for engaging people with lived experience of mental health in research using a wide range of community-based participatory research methods.^54^,^56^ This study emphasised the need to delineate between the use of PPI and citizen science. The delineation of citizen science from other participatory research approaches includes the management of power relations between researchers and citizen scientists. This can be achieved through understanding and reporting the purpose, depth (eg, which parts of the research design cycle) and breadth (eg, the diversity of contributors) of engaging citizen scientists in mental health research. Ensuring that power relations between custodians of knowledge based on their real-world lived experience of mental health challenges (citizen scientists) and researchers are well managed and monitored, and the purpose of knowledge generation and engagement should be clear and towards a common goal, in this instance, the improvement of mental health systems.

Achieving a successful mental health citizen science project and active research engagement is dependent on clear communication, transparency and openness by researchers and the reduction or elimination of research ambiguity. For instance, the use of digitally enabled citizen science approaches may improve research transparency, communications and collaborative workings.^53^ However, digitally enabled citizen science projects could widen health inequalities among people affected by systemic barriers and low socio-economic status.^37^

Stakeholders indicated the potential of a citizen science approach for engaging the public in influencing policy and using their knowledge to improve mental health and health services at a wider level. Our stakeholders stated the importance of co-producing the citizen science infrastructure with the public (*eg, citizen science activity platform)* to promote inclusive knowledge generation and a safe space for critical voices to be heard in mental health research. To encourage co-production and trust building between researchers and citizen scientists, more use of inclusive language in research is required, such as replacing the use of the term *citizen science* with *community science’* Moreover, the term *community science* as an approach is different from citizen science. C*ommunity science* refers to the collective efforts of community groups, experts and specialists who work together to provide scientific solutions at the community level.^57^ It involves community members taking ownership of their environment through data gathering and observational analysis.^57^ Thus, stakeholders suggest retaining the use of *citizen science*, despite its limitations, until a more inclusive terminology emerges.

The transformative potential of using a citizen science approach in mental health research to promote active engagement of the public at different stages of research projects was recognised and acknowledged, especially engaging people who are often not involved in research or mental health systems. This will facilitate the move from research involvement tokenism to more equitable power-sharing and decision-making, promoting empowerment and co-production between stakeholders.^58^ At present, PPI practices can involve a narrow group of individuals in involvement activities, with little consideration for involving broader demographics of the population.^58^ The use of citizen science will promote large-scale public engagement in mental health research beyond data collection, including through greater public knowledge about mental health and the individual and societal changes needed to create better mental health. It may expand the participation of diverse groups of the public in all aspects of research through larger-scale engagement. Care will be needed to ensure equity of access, including for digitally disadvantaged people, particularly given that technological mechanisms such as websites are frequently used in citizen science projects.

Equitable power sharing in decision-making about the research with citizen scientists is required in the development and implementation of mental health research using a citizen science approach. The use of the citizen science approach in mental health should not undermine the impact of PPI over the years in mental health research, but it can encourage changes required to improve ways of working with the public in mental health research. An Australian study showed that the citizen science approach can support and complement existing community engagement approaches, providing a robust engagement framework, rich data access, meaningful engagement and mutual benefits for all stakeholders, including the community members.^59^

### Strengths and limitations

Engaging with the public and generating new mental health knowledge using citizen science is complex and requires resources and planning. Despite the nuances in the use of citizen science in mental health, its use in encouraging co-creation and shared ownership of mental health research works between researchers and the public is feasible, beyond the common practice of public involvement. A strength of this study is the involvement of different stakeholders with diverse knowledge and expertise, including lived experience of mental health challenges, which maximised the applicability of the ToC and will inform the use of citizen science in mental health research. A limitation of the study was not considering the practical implications of the ToC on citizen scientists, human resources, cost and other resources required to set up and implement a mental health citizen science project. The cost-effectiveness of the citizen science approach compared with other participatory research was also not considered. However, this process emphasised the need for synergy between researchers, stakeholders and citizen scientists to ensure active engagement and move beyond research involvement tokenism in mental health research. The development of the ToC focuses on providing information and understanding about how a mental health citizen science project can work, rather than clear guidance on every detail of the project. The ToC was designed as a planning tool to aid the evaluation of mental health citizen science projects rather than as an implementation tool or a comprehensive project guide. Thus, we did not capture any form of challenges that might occur during project implementation. Therefore, a limitation is that researchers and the public may conclude that the identified underpinning assumptions are not accurate. One of the stakeholders stated, “*The ToC is underpinned with assumptions. During the project implementation, we will accurately compare and report the assumptions against the realities of the project*”. Therefore, this ToC map will be refined, and the pathway will be flexible to changes and enhancements as the project progresses.

ToC is a live process and will not end with the production of this publication. Rather, this is a strategy to guide the C-STACS project and will be reviewed and refined during and at the end of the project. Articulating the theory behind the design of research projects is a prerequisite for effective and efficient planning and implementation, as well as planning.^47 48 60^ A ToC is a working document that needs to be updated to reflect learnings and new conditions of the project.^61^

We recommend that researchers designing or implementing a mental health citizen science project continue to share detailed descriptions of their studies. This will illuminate understanding of how the use of citizen science advances mental health practice, given the paucity of literature available on the topic to date.

### Next steps

One motivator for constructing our ToC was to identify how to actively engage the public in the C-STACS project. The next phase of the study will focus on (i) developing two citizen science projects - *Looking after Yourself* and *Envisioning Recovery Support*, (ii) creating a community of mental health-related citizen scientists, (iii) engaging with the citizen scientists, (iv) obtaining inputs from all the stakeholders on the progress of the mental health citizen science project, (v) evaluating the impact of mental health citizen scientist community and process of establishing a mental health citizen science project and (vi) understanding how to engage with people who are digitally excluded, for example, due to low skills or no access to technology equipment. Monitoring the utility of our ToC in C-STACS (and in other projects) will require attention to how to operationalise entities in our ToC, such as the indicators of scale of public involvement that we define.

## Conclusion

The application of the citizen science approach in mental health research is limited and complex. The ToC can be applied to any type of mental health citizen science project to ensure the long-term goal of using the approach to promote active public engagement, mutual benefits between researchers and the public and impact on mental health outcomes. The development of this ToC was supported by seven consultation sessions with stakeholders with mental health and citizen science expertise and knowledge of relevant literature and practices. The agreed impact of a citizen science mental health project is to ensure the co-production of and build the capacity of citizen scientists in mental health research, in this instance, people with lived experience of mental health. This will allow them to influence the social issues that affect their care and mental health outcomes. Stakeholders proposed long-term and short-term outcomes (preconditions) to achieve this impact. The stakeholders in this study highlighted the importance of transparency and openness, context in achieving this impact and the complexity of using a citizen science approach. In conclusion, the use of citizen science in mental health research is achievable, and the assumptions that have been developed through the ToC will be tested in the C-STACS project and evaluated to articulate what works and does not work.

## Supplementary material

10.1136/bmjopen-2024-091007 online supplemental file 1

## Data Availability

All data relevant to the study are included in the article or uploaded as supplementary information.
