# Supplementary material for: How can citizen science enhance mental health research quality: theory of change development
Source: BMJ Open. 2025 Sep 25;15(9):e091007. doi: 10.1136/bmjopen-2024-091007 (PMC12481321; doi:10.1136/bmjopen-2024-091007)
Supplement: online supplemental file 1 [file bmjopen-15-9-s001.docx]

# **Supplemental file: Appendix A -Checklist for reporting ToC in Public Health Interventions**

| Reporting Checklist | Tick/ Explanation |
| --- | --- |
| 1. Is the ToC approach defined? | Yes, PAGE 7 – 8 |
| a. Is a definition of ToC given by the authors? | Yes, PAGE 7 – 8 |
| b. Do the authors explain their reasons for using a ToC approach? | Yes, PAGE 7 – 8 |
| 2. Is the ToC development process described? | Yes, PAGE 7 – 8 |
| a. Are the methods used to develop the ToC, such as stakeholder meetings and interviews, document reviews, programme observation, existing conceptual frameworks or published research, described? | Yes, Table 1 |
| b. Where stakeholders are involved, is it clear how many stakeholders participated, what their role is in relation to the intervention, how they were consulted (e.g. number of interviews, focus groups, ToC workshops) and the extent to which the consultations were participatory? | Yes, Table 1 |
| c. Is the method used to compile the data into a ToC described? (including how disagreements between stakeholders were resolved) | Yes, Table 1 |
| d. Is the extent to which stakeholders were able to validate the resultant ToC and were owners of the final product described? | Yes |
| 3. Is the resultant ToC (or a summary thereof) depicted in a diagrammatic form and does it include? | Ye s, Figure 1 |
| a. The long-term outcome or impact of the intervention | Yes |
| b. The anticipated short and medium-term outcomes and the process of change | Yes |
| c. The intervention components which happen at different stages of the pathway | Yes |
| d. The context of the intervention | Yes |
| e. Assumptions about how change would occur | Yes |
| f. Additional ToC elements such as indicators, supporting research evidence, beneficiaries, actors in the context, sphere of influence and timelines where relevant. | Yes |
| 4. Is the process of intervention development from the ToC described? | Yes |
| a. Are the methods of how interventions were refined from the ToC to something which can be implemented described? (For example, further stakeholder workshops, interviews, systematic literature reviews) | Yes |
| 5. Is the way in which the ToC was used to develop and implement the evaluation described? | No, this si not part of this project phase |
| a. Are evaluation research questions generated from the ToC? | Not applicable, as the project has not been evaluated |
| b. Is the role of ToC in the design, plan or conduct of the evaluation clear? | The role of the Toc is for the design and planning of the project, |
| c. Does the paper describe the extent to which the key elements described in the ToC were measured in the evaluation (i.e., impact, short and medium-term outcomes and the process of change, context, assumptions and the intervention)? | Not applicable, as the project has not been evaluated |
| d. Does the paper describe whether and how process indicators were used to improve the quality of the intervention? | Not applicable, as the project has not been evaluated |
| e. Is the role of the ToC in the analysis of the results of the evaluation clear? | Not applicable, as the project has not been evaluated |
| f. Is the role of ToC in the interpretation of the results of the evaluation described? (Including the breakdown of programme theory, unanticipated outcomes and causation including the strength and direction of causal relationships) | Not applicable, as the project has not been evaluated |

**Supplemental file: Appendix B:** An example of a meeting agenda to facilitate ToC

Citizen Science to Achieve Coproduction at Scale (C-STACS)

Advisory Board Meeting 3

13th July 2023, 10:00–11:00 am

Agenda

Via Microsoft Teams

Welcome and Introductions

1. Project update

- C-STACS citizen science protocol
- Current progress (platforms, projects)
- Project Extension

1. Theory of Change  [Document 1]
2. Any other business
