# Supplementary material for: How can citizen science enhance mental health research quality: theory of change development
Source: BMJ Open. 2025 Sep 25;15(9):e091007. doi: 10.1136/bmjopen-2024-091007 (PMC12481321; doi:10.1136/bmjopen-2024-091007)
Supplement: Supplementary file 2 [file bmjopen-2024-091007.reviewer_comments.pdf]

PEER REVIEW HISTORY

BMJ Open publishes all reviews undertaken for accepted manuscripts. Reviewers are asked to complete a checklist review form and are provided with free text boxes to elaborate on their assessment. These free text comments are reproduced below.

ARTICLE DETAILS

Title (Provisional)

How can citizen science enhance mental health research quality: theory of change development

Authors

Todowede, Olamide; Rennick-Egglestone, Stefan; Boyd, Doreen; Moran, Stuart; Bell, Andy; Sweeney, Angela; Hart, Akiko; Tomlin, André; Robotham, Dan; Repper, Julie; Rimmer, Kara; Brown, Mark; Howells, Mark; Singh, Shuranjeet; Lavis, Paula; Higton, Fred; Hendy, Corrine; Slade, Mike

---

VERSION 1 - REVIEW

**Reviewer**	**1**
**Name**	**Smederevac, Snezana**
**Affiliation**	**University of Novi Sad**
**Date**	**22-Sep-2024**
**COI**	**None**

This article offers a comprehensive overview of how a Theory of Change (ToC) methodology was applied to a mental health citizen science project. The step-by-step process of defining impact, outcomes, and interventions is clearly laid out, with a strong emphasis on stakeholder engagement and iterative consultations. However, the lack of scientifically relevant data analysis and the need for more specific details around outcomes, assumptions, and indicators limit its overall impact.

1. A significant shortcoming is the lack of relevant scientific data analysis. While the section describes reflexive thematic analysis and member-checking, it does not address the analysis of quantitative or qualitative data in a way that meets scientific standards. For instance, the description of the analysis process remains too focused on the iterative consultation framework and lacks rigor in terms of data-driven insights or results. Including a scientifically robust data analysis methodology would enhance the article’s contribution to the field.

2. While the indicators provided are helpful, there is limited detail about how these indicators will be measured or tracked over time. For example, although it is stated that the number of participants and engagement metrics will be tracked, it remains unclear how

demographic diversity or the quality of engagement will be quantitatively or qualitatively assessed. More detailed strategies on how these indicators will be operationalized would strengthen the evaluation framework.

3. The acknowledgment by the stakeholders that influencing policy will take extended time is critical, as policy change is often a long-term process that may not align with short-term project timelines. However, without a specific strategy to actively engage policymakers throughout the project, the notion of influencing policy can remain an aspirational goal rather than a realistic outcome. Simply acknowledging the need for time is insufficient; the article lacks a concrete plan for sustained engagement with policymakers, such as regular policy briefs, stakeholder forums, or participation in advisory committees that could foster long-term relationships with decision-makers. Without such a strategic approach, the goal of policy influence risks becoming a “good wish” rather than a tangible outcome. For instance, there could be a formal mechanism for monitoring and reporting on how project outputs are translated into actionable policy recommendations. Moreover, an assessment of which policies or sectors are most likely to be impacted should be explicitly defined. A roadmap of steps for engaging with policy audiences during and after the project's completion would give the article a more grounded and actionable framework for achieving policy influence, ensuring the project’s impact goes beyond academic and community engagement.

4. While the long-term outcomes mentioned are aligned with the goals of citizen science and mental health research, they remain somewhat vague. Providing clearer definitions or measurable goals (e.g., how increased co-production will manifest or how public engagement will be tracked) would improve the article's practical applicability. The vague framing of these outcomes, without specific benchmarks or examples, makes it difficult to determine how progress towards these goals will be monitored. A more detailed and measurable outcome framework would offer clarity to readers and potential practitioners.

In general, I cannot recommend this article for publication due to the lack of a solid scientific structure. While citizen science is indeed an important and growing aspect of the research landscape, it is crucial to maintain a level of scientific rigor that meets the standards expected for scholarly publications. The current manuscript falls short in this regard.

For a scientific article to make a meaningful contribution, it must go beyond descriptive reporting and demonstrate a rigorous, replicable approach to research. This includes clear methodologies, well-defined data analysis techniques, and measurable outcomes. Without these elements, the article lacks the necessary foundation to be considered a valid scientific contribution.

To ensure that citizen science projects are effectively integrated into the academic discourse, they must adhere to the same standards of rigor and transparency expected of any research endeavor. Simply describing procedures is not enough; a stronger methodological framework, accompanied by relevant data analysis and interpretation, is essential. As it stands, this article does not meet these criteria.

**Reviewer**	**2**
**Name**	**Handley, Melanie**
**Affiliation**	**University of Hertfordshire, CRIPACC**
**Date**	**27-Sep-2024**
**COI**	**None**

Thank you for the opportunity to review this manuscript that presents a co-produced Theory of Change for the use of citizen science in mental health research. This is an useful framework for researchers aiming to increase access and opportunities with the general public in mental health research. The manuscript is well written and my comments are minor.

Mental Health Citizen Science Research Context, Page 9, line 17 should "mental problems" be "mental health problems"?

Discussion

Page 17, lines 1 - 45) The first paragraph of the discussion has several very long sentences. These would benefit from either editing or use of different punctuation.

The statement page 18/19 "The use of citizen science will promote large-scale public engagement in mental health research beyond data collection and expand the participation of diverse groups of the public in all aspects of research." would benefit from some clarification for how (as in the ToC), or linking to how this has been achieved in other disciplines (e.g. environmental and ecological sciences with apps and national volunteering opportunities).

**Reviewer**	**3**
**Name**	**Michel, Janet**
**Affiliation**	**Swiss Tropical and Public Health Institute, Epidemiology**
**Date**	**18-May-2025**
**COI**	**N/A**

This is a very important topic and the paper is written well with regards to grammar and language except for line 10-please review (provided rather)

Line 31 : Be consistent and use either co-production or coproduction

What software was used in data analysis?

Indicators line 46

These are numbers, quantitative in nature and cannot tell the whole story. Authors should also include qualitative indicators, stories of how this engagement changes the lives of people with mental health issues. Secondly, make sure you have a baseline, midline and endline measure of outcome rather than just one data point

See:
https://www.frontiersin.org/journals/epidemiology/articles/10.3389/fepid.2025.1460997/full

Page 15 0f 30 the line numbering starts again at 1-please check

line 3-10 on page 15 : These numbers are activities and outputs-authors need to include outcome indicators even if short-medium outcomes .

Line 14 on page 14 please review the line numbering :The whole section on Preconditions-I miss references in this whole section up to discussion

Summary

I was excited to read this. The methodology section fell short for me. How the theory of change was developed, the process from the begining to the end was not clearly spelt out. The involvement of people with mental health issues is commendable but what is not clear is how many were from a scientific background and what conditions did they suffer from. This is very important because mental health consists of many conditions, Were all conditions represented?

A table of participants could help.

With regards to the data analysis it is not clear whose perpectives were taken into accaount nor is it clear who said what. This flies on the face of involving key-stakeholders. Presentation of the findings according to the groups of stakeholders and how these were incorporated into the ToC and how dievergent views were dealt with etc are missing steps.

At the end the ToC statement is not stated clearly. I suggest the authors use the If we ... and ...then logic and also the diagramatic depiction needs to be updated with a clear flow of how activities lead to outputs, outcomes etc.

The outcomes need to be included even if these are short to medium ones.

I propose a major revision

**** **The reviewer provided a marked copy with additional comments. Please contact the publisher for full details.** ****

VERSION 1 - AUTHOR RESPONSE

Reviewer: 1
1. A significant shortcoming is the lack of relevant scientific data analysis. While the section describes reflexive thematic analysis and member-checking, it does not address the analysis of quantitative or qualitative data in a way that meets scientific standards. For instance, the description of the analysis process remains too focused on the iterative consultation framework and lacks rigor in terms of data-driven insights or results. Including a scientifically robust data analysis methodology would enhance the article's contribution to the field.

Authors: We have updated our methods section to clarify that our Theory of Change was co-produced with the stakeholders of an ongoing citizen mental health process, through a consultation process. This means that the final Theory of Change model should be seen as a co-produced artefact embedding the knowledge and experience of the stakeholders, and hence making that knowledge and experience accessible to the reader, to inform the development of a community of practice around citizen mental health science. Whilst some analysis was conducted after each consultation, for the purpose of feeding information into a subsequent consultation, the emphasis of our work is on reporting the co-production process and on what it produced, rather than on reporting this empirical analysis work. We hope that our methods section now provides a clearer statement of the approach that we took.

2. While the indicators provided are helpful, there is limited detail about how these indicators will be measured or tracked over time. For example, although it is stated that the number of participants and engagement metrics will be tracked, it remains unclear how demographic diversity or the quality of engagement will be quantitatively or qualitatively assessed. More detailed strategies on how these indicators will be operationalized would strengthen the evaluation framework.

Authors: We have refined the names of indicators, to make it clearer as to exactly what characteristics they refer to. We have added text to the "Next steps" section of our discussion to indicate that the indicators will need to be operationalised to enable the direct evaluation of how change is created by the citizen mental health science project that we are working on. The seven consultations that produced our ToC did not consider the exact mechanisms by which indicators would be operationalised, and hence we cannot report on this topic in our manuscript

3. The acknowledgment by the stakeholders that influencing policy will take extended time is critical, as policy change is often a long-term process that may not align with short-term project timelines. However, without a specific strategy to actively engage policymakers throughout the project, the notion of influencing policy can remain an aspirational goal rather than a realistic outcome. Simply acknowledging the need for time is insufficient; the article lacks a concrete plan for sustained engagement with policymakers, such as regular policy briefs, stakeholder forums, or participation in advisory committees that could foster long-term relationships with decision-makers. Without such a strategic approach, the goal of policy influence risks becoming a "good wish" rather than a tangible outcome. For instance, there could be a formal mechanism for monitoring and reporting on how project outputs are translated into actionable policy recommendations. Moreover, an assessment of which policies or sectors are most likely to be impacted should be explicitly defined. A roadmap of steps for engaging with policy audiences during and after the project's completion would give the article a more grounded and actionable framework for achieving policy influence, ensuring the project's impact goes beyond academic and community engagement.

Authors: We agree with the reviewer's argument here. However, as for indicators, our consultations did not cover the exact process by which policy could be changed, and hence, we cannot report on this. We have updated our text to acknowledge that policy change is not just a feature of time taken, but requires specific planning.

4. While the long-term outcomes mentioned are aligned with the goals of citizen science and mental health research, they remain somewhat vague. Providing clearer definitions or measurable goals (e.g., how increased co-production will manifest or how public engagement will be tracked) would improve the article's practical applicability. The vague framing of these outcomes, without specific benchmarks or examples, makes it difficult to determine how progress towards these goals will be monitored. A more detailed and measurable outcome framework would offer clarity to readers and potential practitioners.

Authors: We have revised the names and descriptions of all entities in the Theory of Change, to make them more specific and clearer, including through providing examples in our Results section to elucidate them.

Reviewer: 2

Discussion
Page 17, lines 1 - 45) The first paragraph of the discussion has several very long sentences. These would benefit from either editing or use of different punctuation.

Authors: We have thoroughly revised the discussion, adding structure and breaking it up into more paragraph

The statement page 18/19 "The use of citizen science will promote large-scale public engagement in mental health research beyond data collection and expand the participation of diverse groups of the public in all aspects of research." would benefit from some clarification for how (as in the ToC), or linking to how this has been achieved in other disciplines (e.g. environmental and ecological sciences with apps and national volunteering opportunities).

Authors: We have clarified this statement, thank you.

Reviewer: 3 (Please see also pdf attached with their comments in your manuscript)
This is a very important topic and the paper is written well with regards to grammar and language except for line 10-please review (provided rather)

Authors: Done

Line 31 : Be consistent and use either co-production or coproduction

Authors: We have standardised on coproduction.

What software was used in data analysis?

Authors: NVIVO was used. However, we have thoroughly updated our methods section to draw attention to the overall structure of our coproduction process, through which our ToC was developed. Whilst there was analysis work conducted after each consultation, to prepare for the subsequent consultation, then most of the work of developing the ToC was done in the consultation sessions, and through discussion between the researchers in between sessions.

This has meant that we have chosen not to foreground analysis work, since it was just a small part of our process. We are, of course, willing to revise this part of our paper if needed.

Indicators line 46
These are numbers, quantitative in nature and cannot tell the whole story. Authors should also include qualitative indicators, stories of how this engagement changes the lives of people with mental health issues. Secondly, make sure you have a baseline, midline and endline measure of outcome rather than just one data point
See:
https://www.frontiersin.org/journals/epidemiology/articles/10.3389/fepid.2025.1460997/full

Page 15 0f 30 the line numbering starts again at 1-please check
line 3-10 on page 15 : These numbers are activities and outputs-authors need to include outcome indicators even if short-medium outcomes .

Authors: We have thoroughly revised our diagram and text so that they match

Line 14 on page 14 please review the line numbering :The whole section on Preconditions-I miss references in this whole section up to discussion

Summary
I was excited to read this. The methodology section fell short for me. How the theory of change was developed, the process from the begining to the end was not clearly spelt out. The involvement of people with mental health issues is commendable but what is not clear is how many were from a scientific background and what conditions did they suffer from. This is very important because mental health consists of many conditions, Were all conditions represented? A table of participants could help.

Authors: Thank you. We have thoroughly revised our methods section from start to end, with the intention of clarifying our whole process, and the people who were involved. We now justify our choice of iterative consultation, which we felt was appropriate given the very small number of completed citizen mental health studies. We have descried the relevant experience of the stakeholders in more specific detail, including providing a table describing the public organisations from whom representatives were drawn. We did not ask for precise details from each individual of mental health experience, but we can be confident that the stakeholder team include mental health experience of mood disorders (including bipolar disorders), psychosis, and personality disorders.

With regards to the data analysis it is not clear whose perpectives were taken into accaount nor is it clear who said what. This flies on the face of involving key-stakeholders. Presentation of the findings according to the groups of stakeholders and how these were incorporated into the ToC and how dievergent views were dealt with etc are missing steps.

Authors: We have updated our methods section to explain our consultations in more detail. Essentially, the ToC map was coproduced during consultation meetings, rather than outside of them. This means that stakeholders examined prototype versions of the ToC in meetings, and agreed together how to refine them in meetings. This means that we cannot provide information on the perspectives that individual stakeholders brought, because stakeholders refined the map together, in a collaborative process.

At the end the ToC statement is not stated clearly. I suggest the authors use the If we … and …then logic and also the diagramatic depiction needs to be updated with a clear flow of how activities lead to outputs, outcomes etc. The outcomes need to be included even if these are short to medium ones.

Author: Whilst we have refined our ToC for clarity, we can only report on what was developed during consultations, since coproduction was an essential part of our process.
